# Engraftment of self-renewing endometrial epithelial organoids promotes endometrial regeneration by differentiating into functional glands in rats

**DOI:** 10.3389/fbioe.2024.1449955

**Published:** 2024-12-11

**Authors:** Yana Ma, Jingjing Qian, Xin Xu, Cheng Wei, Minyuan Wang, Peipei Zhang, Sijia Chen, Lingyan Zhang, Yanling Zhang, Yanpeng Wang, Wenzhi Xu, Mengying Liu, Xiaona Lin

**Affiliations:** ^1^ Assisted Reproduction Unit, Department of Obstetrics and Gynecology, Sir Run Run Shaw Hospital, Zhejiang University School of Medicine, Hangzhou, Zhejiang, China; ^2^ Zhejiang Key Laboratory of Precise Protection and Promotion of Fertility, Hangzhou, China; ^3^ Department of Obstetrics and Gynecology, Yuyao People’s Hospital of Zhejiang Province, Ningbo, China; ^4^ Zhejiang Provincial Clinical Research Center for Obstetrics and Gynecology, Hangzhou, China; ^5^ Department of Gynecology, Wenling First People’s Hospital of Zhejiang Province, Taizhou, China; ^6^ Department of Obstetrics and Gynecology, Tiantai People’s Hospital of Zhejiang Province, Taizhou, China; ^7^ Center for Reproductive Medicine, Department of Gynecology, Zhejiang Provincial People’s Hospital, Affiliated People’s Hospital, Hangzhou Medical College, Hangzhou, China

**Keywords:** endometrial regeneration, 3D-culture, endometrial epithelial organoids, bone marrow mesenchymal stem cells, transplantation, infertility

## Abstract

**Introduction:**

Extensive trauma frequently disrupts endometrial regeneration by diminishing endometrial stem cells/progenitor cells, affecting female fertility. While bone marrow mesenchymal stem cell (BMSC) transplantation has been suggested as an approach to address endometrial injury, it comes with certain limitations. Recent advancements in endometrial epithelial organoids (EEOs) have displayed encouraging potential for endometrial regeneration. Therefore, this study aims to explore whether EEOs surpass BMSCs in their ability to repair injured endometrium and to examine whether the restoration process involves the integration of EEOs into the endometrial tissue of the recipient.

**Methods:**

We developed rat EEOs (rEEOs) mimicking the features of the rat endometrium. Subsequently, we created a rat model of endometrial injury to compare the effects of rEEOs and rat BMSCs (rBMSCs) on endometrial regeneration and reproductive recovery. Bulk RNA-sequencing analysis was conducted to further investigate the capacity of rEEOs for endometrial regeneration and to identify discrepancies between rEEOs and rBMSCs. Additionally, to track the fate of the transplanted cells *in vivo*, we transplanted green fluorescent protein (GFP) -labelled rEEOs or red fluorescent protein (RFP) -labelled rBMSCs.

**Results:**

In a rat model of endometrial injury, we observed that fertility recovery in rats transplanted with rEEOs was more comparable to that of normal rats than in those treated with rBMSC. rEEOs possess a high concentration of endometrial epithelial stem/progenitor cells and secrete vascular endothelial growth factor (VEGF)-A to promote endometrial neovascularization. Significantly, we observed that cells from GFP-labelled rEEOs could integrate and differentiate into functional glands within the injured endometrium of recipient rats.

**Discussion:**

EEOs offer a transformative approach to address the challenges of endometrial trauma. Their remarkable regenerative potential holds promise for the restoration of damaged endometrium. As we venture into the future, the concept of utilizing patient-specific EEOs for transplantation emerges as a tantalizing prospect. However, the EEOs in our experiments were mainly cultured in Matrigel, which has barriers to clinical translation as a biomaterial, a new biomaterial to be explored. Secondly, our experiments have been successful only in rat models, and more efforts need to be made before clinical translation.

## Introduction

Periodic shedding, regeneration, and remodeling of the human endometrium, orchestrated by estrogen and progesterone, form the foundation for successful embryo implantation in the female reproductive system (Critchley et al., 2020; Evans et al., 2016). However, certain traumatic surgeries, such as repetitive curettage and hysteroscopy, can damage the endometrial basal layer and frequently disrupt endometrial regeneration, resulting in severe conditions, such as thin endometrium and intrauterine adhesions (IUA) (Lee et al., 2021; Yu et al., 2008). Impaired endometrial regeneration is identified by gland inactivity, limited interstitial cells, and inadequate vascularization, substantially affecting female fertility (Yu et al., 2008). A meta-analysis showed that approximately 19% of women experiencing miscarriages were diagnosed with IUA within 12 months (Hooker et al., 2014). Restoring epithelial tissue is pivotal in preserving endometrial equilibrium after exposure to environmental stress (Gargett et al., 2012; Gargett et al., 2016; Jin, 2019; Salamonsen et al., 2021). Epithelial stem or progenitor cell reserves in the glands and luminal crypts can transform into glandular cells, aiding in endometrial repair following injury (Jin, 2019; Seishima et al., 2019; Syed et al., 2020). Consequently, revitalizing the activity of these endometrial epithelial stem or progenitor cells is a critical focus in enhancing endometrial regeneration and restoring fertility.

Recently, stem and progenitor cell therapies have been used to regenerate the endometrium. Bone marrow mesenchymal stem cells (BMSCs) are frequently used seed cells, demonstrating the potential to enhance fertility (Azizi et al., 2018; Cousins et al., 2021a; Song et al., 2021). Fertility is enhanced by promoting angiogenesis and suppressing endometrial fibrosis through their paracrine effects. However, the potential of BMSCs to differentiate into endometrial cells to repair damaged endometrium remains debated (Cervelló et al., 2015; Liu et al., 2018; Ong et al., 2018; Xiao et al., 2019). Safety concerns, preservation complexities, and immune rejection pose substantial barriers to clinically using BMSCs (Azizi et al., 2018; Cousins et al., 2021a; Song et al., 2021). Therefore, developing novel clinical treatments to address endometrial injury can enhance reproductive outcomes.

3D-cultured organoids originating from stem cells or organ progenitors are increasingly used to treat organ injuries (Lancaster and Knoblich, 2014). Organoid transplantation exhibits substantial promise in the repair of various organs, including the colon (Yui et al., 2012; Fordham et al., 2013; Sugimoto et al., 2018; Jee et al., 2021), brain (Revah et al., 2022; Wilson et al., 2022; Jgamadze et al., 2023; Zheng et al., 2023), retina (McLelland et al., 2018), and lungs (Louie et al., 2022), by seamlessly integrating at the injury site. In reproductive medicine, endometrial epithelial organoids (EEOs) derived from endometrial glandular fragments that successfully mirror the crucial characteristics of the uterine epithelium have been developed (Boretto et al., 2017; Turco et al., 2017). Endometrial organoids derived from mouse and human embryonic stem cells promote endometrial regeneration (Jiang et al., 2021; Zhang et al., 2022). Moreover, a co-culture system was designed to generate multi-lineage endometrial organoids comprising EEOs and endometrial mesenchymal stem cells that repaired endometrial damage (Xu et al., 2023).

However, additional research is needed to explore how EEOs repair injured endometrial tissues and ascertain their potential advantages over BMSCs. Therefore, this study aimed to investigate the comparative capacity of EEOs and BMSCs in repairing impaired endometrium and examine integrating EEOs into the endometrial tissue of the recipient during endometrial restoration.

## Materials and methods

### Animals

Sprague–Dawley (SD) rats (5–8 weeks old) were obtained from the Zhejiang Academy of Medical Sciences (China). Green fluorescent protein (GFP)-labeled SD rats were obtained from the Zhejiang Animal Center (China). The rats were housed in a controlled environment at 22°C with a 12-h light/dark cycle. A 1-week acclimation period was allowed before the experiments. To reduce the impact of the sexual cycle on endometrial restoration, the rats were subcutaneously injected with estrogen (E2, 1 µg/200 g, Beyotime, Shanghai, China, ST1101) for three consecutive days to confirm that they were in the oestrus phase before surgery. The Animal Ethics Committee of Zhejiang University approved all animal experiments (approval number: ZJU20220453; date of approval: 2 December 2022).

### Rat EEOs isolation and cultivation

The method used to isolate endometrial fragments was adapted from a protocol designed for mouse glandular fragments (De Clercq et al., 2017). Female SD rats (5 weeks old) and GFP-labeled rats (5 weeks old) were euthanized with carbon dioxide, and their uteri were longitudinally opened and dissected into 1 cm fragments. The fragments were digested in a solution comprising 1× phosphate-buffered saline (PBS) supplemented with 0.5% pancreatin (Sigma-Aldrich, St. Louis, MO, United States, P3292) and 0.25% trypsin (Sigma-Aldrich, T7409). Digestion involved shaking at 4°C for 60 min, followed by incubation for 45 min at 23°C and 15 min at 37°C without shaking. After digestion, the uteri were transferred into a 15 mL tube containing 3 mL of cold PBS after terminating the process with fetal bovine serum (FBS, Cellmax, Beijing, China, SA112.02). The epithelial sheets were released by vortexing for 30 s. This process was repeated five times to obtain five suspensions containing epithelial sheets. Tissue debris was removed by filtration using a 100 μm cell sieve (Corning, NY, United States, 352360). The collected filtrate was then passed through a 40 μm cell sieve (Corning, United States, 352340) to eliminate stromal cells. Subsequently, the residual glandular materials were rinsed from the sieve membranes by inverting the 40 μm sieve. The obtained glandular material was centrifuged at 600 *g* for 5 min and resuspended in a solution containing ice-cold 70% Matrigel (Corning, 356231) and 30% Dulbecco’s Modified Eagle’s Medium (DMEM)/F12 (Meilunbio, Dalian, China, PWL005). Matrigel-cell suspension (25 μL) was plated in each well of a 48-well plate. Upon solidification of the Matrigel, 250 μL of organoid Expansion Medium (ExM) was added. The ExM comprised DMEM/F12 supplemented with B27, N2, insulin-transferrin-selenium, nicotinamide, epidermal growth factor, fibroblast growth factor 10, noggin, transforming growth factor beta/Alk inhibitor A83-01, ROCK inhibitor Y27632, and Wnt activators Wnt3a and R-Spondin-1 (Supplementary Table S1). Rat EEOs (rEEOs) with low passage numbers (3–5 passages) were used for the experiments. Female SD rats or rEEOs derived from these rats were used in this study, except for the experiment to confirm the successful injection of GFP-labeled rEEOs and RFP-labeled rBMSCs in which GFP-labeled rats were used.

### Cultivation and passaging of rat BMSCs

Red fluorescent protein (RFP)-labeled rat BMSCs (rBMSCs) were obtained from Cyagen Biosciences Inc. (RASMX-01201, Guangzhou, China). After thawed in a 37°C water bath, the cells were transferred to a 15 mL centrifuge tube and 9 mL of culture medium was added. Centrifuge at 800 rpm for 5 min to collect cell sediment and then resuspended in the culture medium. Transfer the cell suspension gently into a 6 cm dish and mix well. Place it in a 37°C, 5% CO_2_ incubator for cultivation. The medium was changed every 2 days.

The cells were subcultured in quadruplicates upon reaching 80%–100% confluence. The culture medium was discarded, and the cells were washed once with PBS, 1 mL trypsin (Solarbio, T1300) was added and the cells were incubated at 37°C for 5 min for digestion. Terminate digestion with 2 mL culture medium. Centrifuge at 800 rpm for 5 min to collect cell sediment and then resuspended in the culture medium. Continue the cultivation according to the above method. The culture medium comprised DMEM/F12 supplemented with 1% penicillin-streptomycin solution (Biosharp, BL505A) and 10% (v/v) FBS.

### Hormonal treatment of rEEOs

rEEOs were cultivated for 4 days using phenol red-free ExM to simulate hormonal conditions. The rEEOs were then treated for two consecutive days with E2 at a concentration of 10 nM. The cells were then treated for 4 days with progesterone (P4, Sigma Aldrich, P8783) at a concentration of 1 μM, combined with cyclic adenosine monophosphate (cAMP, MCE, NJ, United States, HY-12306) at a concentration of 1 µM. The culture medium was changed every 2 days.

### Cytohistological analysis

Paraffin and frozen sections were prepared as described by Zhang et al. (2021) to conduct the cytohistological analysis of rEEOs. Uterus specimens were initially fixed in 4% paraformaldehyde at 4°C for 24 h, dehydrated using alcohol, cleared with xylene, and embedded in paraffin. Additionally, fresh uterine tissue was embedded in the optimal cutting temperature compound (OCT, SAKURA, Shanghai, China, 4583) to prepare frozen sections. Immunohistochemistry (IHC) was conducted on paraffin sections by first dewaxing the slices, followed by antigen retrieval through hyperbaric heating. The sections were then incubated overnight at 4°C with primary antibodies, including Ki67 (Abcam, Cambridge, United Kingdom, 1:500), PANCK (Abcam, 1:200), and CD31 (Abcam, 1:4000). The antigens were visualized using a goat anti-mouse/rabbit secondary antibody (Genetech, Shanghai, China, GK500710).

Immunofluorescence (IF) staining was conducted on paraffin-embedded sections using primary antibodies targeting SSEA-1 (Santa Cruz Biotechnology, Dallas, TX, United States 1:200), N-CAD (Cell Signaling Technology, Danvers, MA, United States, 1:400), and GFP (ABclonal, Wuhan, China, 1:50). IF staining was performed on the frozen sections for E-CAD (Cell Signaling Technology, 1:200), FOXA2 (Abcam, 1:300), MUC1 (Abcam, 1:200), VIM (Cell Signaling Technology, 1:200), and SOX9 (Abcam, 1:200). Secondary antibodies conjugated with goat anti-rabbit/mouse IgG secondary antibody Alexa-FluorTM 488/568 (Thermo Fisher Scientific, Waltham, MA, United States, 1:200) were used. The images were analyzed using a confocal microscope (Zeiss LSM800, Oberkochen, Germany).

For histological staining, hematoxylin–eosin (HE) (Beyotime, Jiangsu, China, C0105M) and periodic acid–Schiff (PAS) (Beyotime, C0142M) staining were performed following the manufacturer’s instructions. Additionally, Masson’s trichrome (Masson) staining was conducted according to conventional protocols.

### Enzyme-linked immunosorbent assay

The supernatants of rEEOs and rBMSCs were collected alongside the supernatants of ExM + Matrigel and the culture medium of rBMSCs serving as controls. The supernatants were centrifuged at 1,000 *g* for 10 min at 4°C to eliminate cellular debris. Analysis was conducted using the Rat Vascular Endothelial Cell Growth Factor A (VEGF-A) enzyme-linked immunosorbent assay (ELISA) kit (Elabscience, Wuhan, China, E-EL-R2603c) following the manufacturer’s instructions. Absorbance readings of the samples were measured at 450 nm using an ELX800 Universal Microplate Reader. Concentrations were quantified and expressed as pg/mL.

### Establishment of the endometrial injury model and cell implantation

Ninety-six female SD rats (8 weeks old, 200–230 g) were used for the *in vivo* endometrial repair experiments. An endometrial injury model was created by modifying the colonic injury method (Sugimoto et al., 2018). After 1-week acclimation, the rats were subcutaneously injected with E2 to confirm that they were in the oestrus phase before surgery. All rat modeling is performed by the same operator. Rats were anesthetized with 1.25% tribromoethanol (25 mL/kg, Meilunbio, MA0478), and the abdominal cavity was opened to expose the uterus under aseptic conditions. Arterial clamps were placed at both ends of the uterine horn. Subsequently, the uterine cavity was perfused with 50 µL of 50°C 250 mM EDTA/PBS (Sangon Biotech, Shanghai, China, B540625; Meilunbio, MA0010) for 3 min. The uterine cavity was then gently scratched using a scraper resulting in uterine congestion. The scraping is stopped when the uterine walls transition from smooth to rough, approximately 30 times (Supplementary Figure S3A). The right uterine horn of each rat underwent endometrial scraping, whereas the contralateral left uterine horn was used as a control. The rats with endometrial injury were randomly divided into four groups: Injury group (n = 24), in which the injured right uterine horns received PBS; Matrigel group (n = 24), injured right uterine horns were treated with Matrigel solution; rBMSCs group (n = 24), in which the injured right uterine horn was treated with a suspension of rBMSCs/Matrigel; and the rEEOs group (n = 24), the injured right uterine horn received a suspension of rEEO/Matrigel. Each group received a 50 µL suspension of cells (containing 1 × 10^7^ cells) or Matrigel, implanted carefully into the injured uterine cavity using a 100 µL pipette. The mixture was allowed to solidify within the cavity for 1 min. Rats were euthanized with CO_2_ at 7, 14, and 28 days post-transplantation, and the uterine horns were collected for further analysis. Histological assessments were conducted using HE staining, Masson staining, and IHC (PANCK and CD31) to examine tissue repair evidence.

### RNA-sequencing

Total RNA was extracted from rEEOs (n = 3) and rBMSCs (n = 3) using the RNA-Quick Purification Kit (ES Science, Shanghai, China, RN001) following the manufacturer’s instructions. The purified RNA underwent 150-bp single-end RNA-sequencing (RNA-seq) using the BGISEQ platform (China, BGI). Raw RNA-seq data underwent quality control using TrimGalore to obtain clean data. The cleaned sequence reads were aligned against the *Rattus norvegicus* genome using Bowtie2 (version 2.2.5) (Langmead and Salzberg, 2012).

### RNA-seq data analysis

RNA-seq data was analyzed using the Dr. Tom multi-omics data analysis system (https://biosys.bgi.com). Differentially expressed genes were normalized to transcripts per kilobase of exon model per million mapped reads (TPM) using RSEM (version 1.3.1) (Li and Dewey, 2011). Heatmap clustering of gene expression across samples was analyzed using pheatmap (version 1.0.8). Differential gene detection was performed using DESeq2 (version 1.4.*5*) (Love et al., 2014) with *q* < 0.05. Gene Ontology (GO) enrichment analysis (http://www.geneontology.org/) of differential genes was performed using Phyper. GO terms with corrected *p* < 0.05 were considered significantly enriched. Gene Set Enrichment Analysis (GSEA) was conducted on the official GSEA website (https://www.gsea.org/).

### Fluoroscopic imaging

Fluoroscopic images of the recipient uterus were captured using the IVIS^®^ Lumina LT *In Vivo* Imaging System (PerkinElmer, Waltham, MA, United States). The rats underwent unilateral uterine horn injury according to the previously described modeling method. Subsequently, fluorescently labeled rEEOs and rBMSCs were transplanted into the injured right uterine horn, whereas the contralateral left uterine horns served as controls. One day post-transplantation, the rats were euthanized, and both uterine horns were dissected. Fluorescence images were acquired using Lumina II Living image software (version 4.2).

### Tracing rEEOs and rBMSCs *in vivo*

GFP-labeled rEEOs were generated from GFP-labeled SD rats as described in “Rat EEOs isolation and cultivation” to monitor the fate of the transplanted cells *in vivo*. These GFP-labeled rEEOs and RFP-labeled rBMSCs were transplanted into the injured uterine cavity as previously detailed. After EDTA insufflation, the procedure involved scratching around the uterus and cell transplantation. The right uterine horns were subjected to endometrial damage and cell transplantation, whereas the contralateral left uterine horns were used as controls. Recipient rats received daily subcutaneous injections of sandimmun (2 mg/200 g, NOVARTIS, Basel, Switzerland). Uterine horns were harvested at 7, 14, and 28 days post-transplantation. Frozen sections were used to evaluate the localization and behavior of the transplanted cells *in vivo*. IF staining for E-CAD was conducted as previously outlined to label the epithelium.

### Fertility test

At 28 days post-transplantation, 32 rats (each group contained 8 rats) were paired with male SD rats in a 1:2 ratio to evaluate fertility. The day of observing the vaginal pessary signified day 0 of pregnancy. Upon reaching day 18 of gestation, the rats were euthanized, and the uterus was examined to count the number of embryos on both sides.

### Statistical analysis

ImageJ software (NIH, Bethesda, MD, United States) was used for the histological measurements of the rat uterus, including endometrial thickness (vertical distance between luminal epithelium and circular smooth muscle) (HE staining), percentage of fibrous area (proportion of blue area to total endometrial area) (Masson staining), percentage of positive area of PANCK (proportion of DAB-positive area to total endometrial area) (PANCK staining) and the number of vessels (number of vessels in 400× field of view) (CD31 staining). Three values were averaged for each cross-section, and the mean of the upper, middle, and lower cross-section was calculated for each uterine horn. The Shapiro–Wilk test was used to examine the Gaussian distribution. The independent-sample *t*-test (Normal distribution) and Mann–Whitney *U* test (Non-normal distribution) were used to determine differences between the two groups, one-way analysis of variance (ANOVA) with Bonferroni correction was used for multiple comparisons, and the chi-square test and Fisher’s precision probability test were used to compare pregnancy rates [GraphPad Prism version 8 (La Jolla, CA, United States)]. Statistical significance was set at *p* < 0.05. **p < 0.001, ****p < 0.0001, ns, no significance.

## Results

### rEEOs recapitulate endometrial morphology and function *in vitro*

**FIGURE 1:**
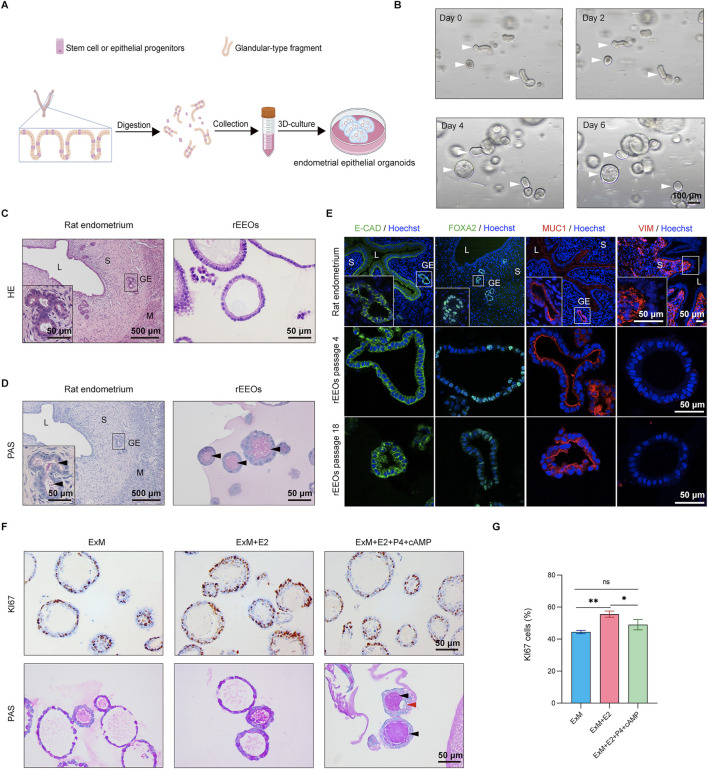
Establishment of rat endometrial epithelial organoids *in vitro*. **(A)** Schematic representation illustrating the derivation process of rEEOs. **(B)** Representative images depicting the progressive growth of rEEOs at various time points. White arrows indicate the self-organization of glands into rEEOs. **(C–D)**. HE and PAS staining comparison between native rat endometrium and rEEOs. Insets display enlarged views of specific areas marked with black squares. **(E)** IF staining of E-CAD, FOXA2, MUC1, and VIM in rat endometrium and rEEOs. Insets display enlarged views of specific areas marked with white squares. **(F)** IHC staining for Ki67 and PAS staining in rEEOs exposed to diverse hormone stimulation conditions. Black arrows indicate mucus secretion by both glands and rEEOs, whereas red arrows indicate the presence of subnuclear vacuolar structures. **(G)** Quantification of Ki67^+^ cells in rEEOs (n = 3). Results are displayed as the mean ± standard deviation (SD) and were compared using one-way ANOVA with Bonferroni correction. L, lumen; S, stroma; GE, glandular epithelium; M, muscular layer.

To generate rEEOs, we isolated endometrial glandular-type fragments from the rat endometrium, which was abundant in epithelial stem/progenitor cells (Gargett et al., 2016; Cousins et al., 2021b). These fragments were then cultivated in Matrigel droplets using ExM containing various nutrients and cytokines under 3D conditions (Figure 1A; Supplementary Table S1). Glandular-type fragments progressively self-organized into multicellular spheroids (Figure 1B).

HE staining revealed that the gross morphology of rEEOs closely resembled that of rat endometrial glands (Figure 1C). Additionally, PAS staining, which detected secretory activity, verified that the rEEOs exhibited secretory functions similar to those observed in the endometrial glands of a native rat (Figure 1D). Endometrial epithelial cell-specific markers, such as E-CAD, FOXA2, and MUC1, were expressed in rEEOs, whereas the stromal cell marker VIM was not (Figure 1E). This expression pattern was comparable to that of native rat endometrial glands, further confirming the similarity between rEEOs and endometrial tissues. Furthermore, the 3D structure and expression of glandular epithelial markers in rEEOs remained consistent across several passages (Figure 1E; Supplementary Figure S1A). rEEOs could also endure freeze-thaw cycles (Supplementary Figure S1B). The stability of this structure and molecular characteristics over an extended period highlights the robustness and reliability of rEEOs as models for investigating endometrial biology (Figure 1E; Supplementary Figure S1A, B). These findings suggest that rEEOs accurately replicate the morphological features of endometrial glands *in vivo* and maintain stability during prolonged *in vitro* culture.

The endometrium undergoes a cyclic transformation in response to ovarian steroid hormones, where estrogen promotes endometrial tissue growth and proliferation, whereas progesterone regulates differentiation and secretory maturation (Roy and Matzuk, 2011). rEEOs were initially subjected to E2 treatment, followed by P4 and cAMP exposure to evaluate the responsiveness of rEEOs to ovarian steroid hormones (Supplementary Figure S2A). As anticipated, E2 treatment increased the number of Ki67^+^ proliferating cells within the rEEOs (Figures 1F, G). Conversely, after P4 treatment, a slight decrease in the number of Ki67^+^ cells was observed, indicating the inhibitory effect of P4 on E2-induced proliferation (Figures 1F, G). P4 and cAMP treatments induced markedly morphological changes in rEEOs, causing wall folding and wrinkling (Supplementary Figure S2B) that lead to sub-nuclear vacuole formation (Figure 1F). Furthermore, the induction of mucin secretion, identified by PAS staining, indicated that P4 enhanced the secretory function of rEEOs. These hormone-induced changes align with previous findings (Boretto et al., 2017) and reflect the physiological function of the endometrium *in vivo*. These findings demonstrate that rEEOs exhibit hormone-responsive characteristics similar to that of native endometrium.

### rEEO transplantation restores injured endometrium in rats

**FIGURE 2:**
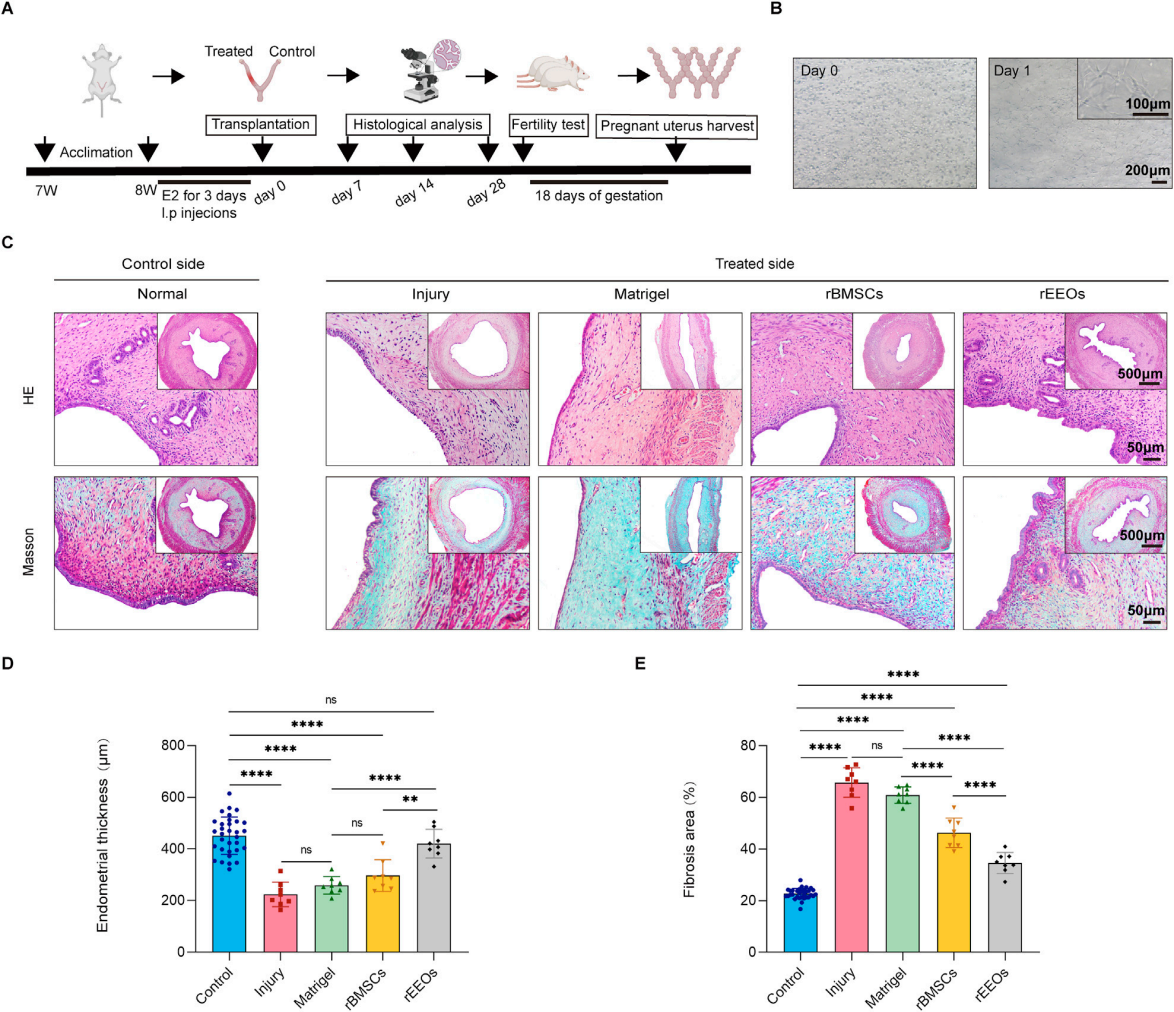
Recovery of endometrial morphology after 28 days of various treatments. **(A)** Experimental protocols for *in vivo* experiments. **(B)** Illustrative images displaying rBMSCs cultivated in 3D-culture with Matrigel. **(C)** HE and Masson staining of consecutive sections of the uterus after 28 days of distinct treatments. Insets display overview images at lower magnification. **(D–E)** Statistical analysis of the normalized changes in endometrial thickness and fibrosis area after 28 days of diverse treatments. Control group (n = 32), Injury group (n = 8), Matrigel group (n = 8), rBMSCs group (n = 8) and rEEOs group (n = 8). The data are presented as the mean ± SD and analyzed using one-way ANOVA with Bonferroni correction.

Treated rat uterine horns were collected and analyzed to assess the therapeutic efficacy of rEEOs in repairing injured endometrium and evaluating the effectiveness of rEEOs compared to that of rBMSCs at various time points (Early: 7 days; Mid: 14 days; Late: 28 days) (Figure 2A). The rat uterine horns were divided into Control group (no injury), Injury group (PBS transplantation after injury), Matrigel group (Matrigel transplantation after injury), rBMSCs group (rBMSCs/Matrigel transplantation after injury) and rEEOs group (rEEOs/Matrigel transplantation after injury). Since the cells were transplanted with Matrigel, we further confirmed that rBMSCs could survive and grow normally in Matrigel (Figure 2B).

Gross inspection of the uteri revealed moderate stenosis and hydrometra in the Injury group, beginning 14 days after transplantation (Supplementary Figure S3B). Conversely, rEEO and rBMSC transplantation alleviated uterine stenosis and hydrops, whereas Matrigel transplantation did not (Supplementary Figure S3B). We next analyzed the thickness of the endometrium in each group of uterine horns. HE staining revealed a markedly reduction in endometrial thickness in the Injury group (Early, 284.6 ± 44.2 μm; Mid, 216.0 ± 32.6 μm; Late, 223.7 ± 47.7 μm) and Matrigel group (Early, 299.9 ± 48.0 μm; Mid, 236.7 ± 47.4 μm; Late, 259.0 ± 33.9 μm) at 7, 14, and 28 days post-injury compared to that of the Control group (Early, 429.9 ± 68.0 μm; Mid, 422.1 ± 56.5 μm; Late, 451.2 ± 72.5 μm) (Figures 2C, D; Supplementary Figure S4A, B; Supplementary Figure S5A, B). In contrast, on day 14 and 28, the rEEOs group (Mid, 414.6 ± 83.9 μm; Late, 420.7 ± 55.6 μm) exhibited a well-organized endometrium with a significant increase in endometrial thickness. Importantly, the endometrial thickness in the rEEOs group was significantly thicker compared to the rBMSCs group (Mid, 317.1 ± 69.2 μm; Late, 297.2 ± 61.3 μm) (Figures 2C, D; Supplementary Figure S5A, B). Endometrial injury is often accompanied by severe collagen deposition and fibrosis. On day 7, Masson staining revealed collagen deposition in all groups following endometrial injury, indicating a consistent presence regardless of the treatment method (Supplementary Figure S4A, C). On days 14 and 28, the Injury group (Mid, 61.5% ± 8.3%; Late, 65.8% ± 5.7%) and Matrigel group (Mid, 55.6% ± 3.6%; Late, 60.9% ± 3.2%) showed severe collagen deposition compared to the Control group (Mid, 21.5% ± 1.6%; Late, 22.7% ± 2.1%), whereas the rEEOs group (Mid, 36.3% ± 2.4%; Late, 34.6% ± 4.1%) and rBMSCs group (Mid, 41.2% ± 4.4%; Late, 46.3% ± 5.8%) exhibited mild deposition (Figures 2C, E; Supplementary Figure S5A, C). The rEEOs group demonstrated significantly weaker collagen deposition than the rBMSCs group on day 28 (Figures 2C, E). These findings suggest that transplanting rEEOs offers advantages over rBMSCs in enhancing the injured endometrium in regards to endometrial thickness and collagen deposition.

**FIGURE 3:**
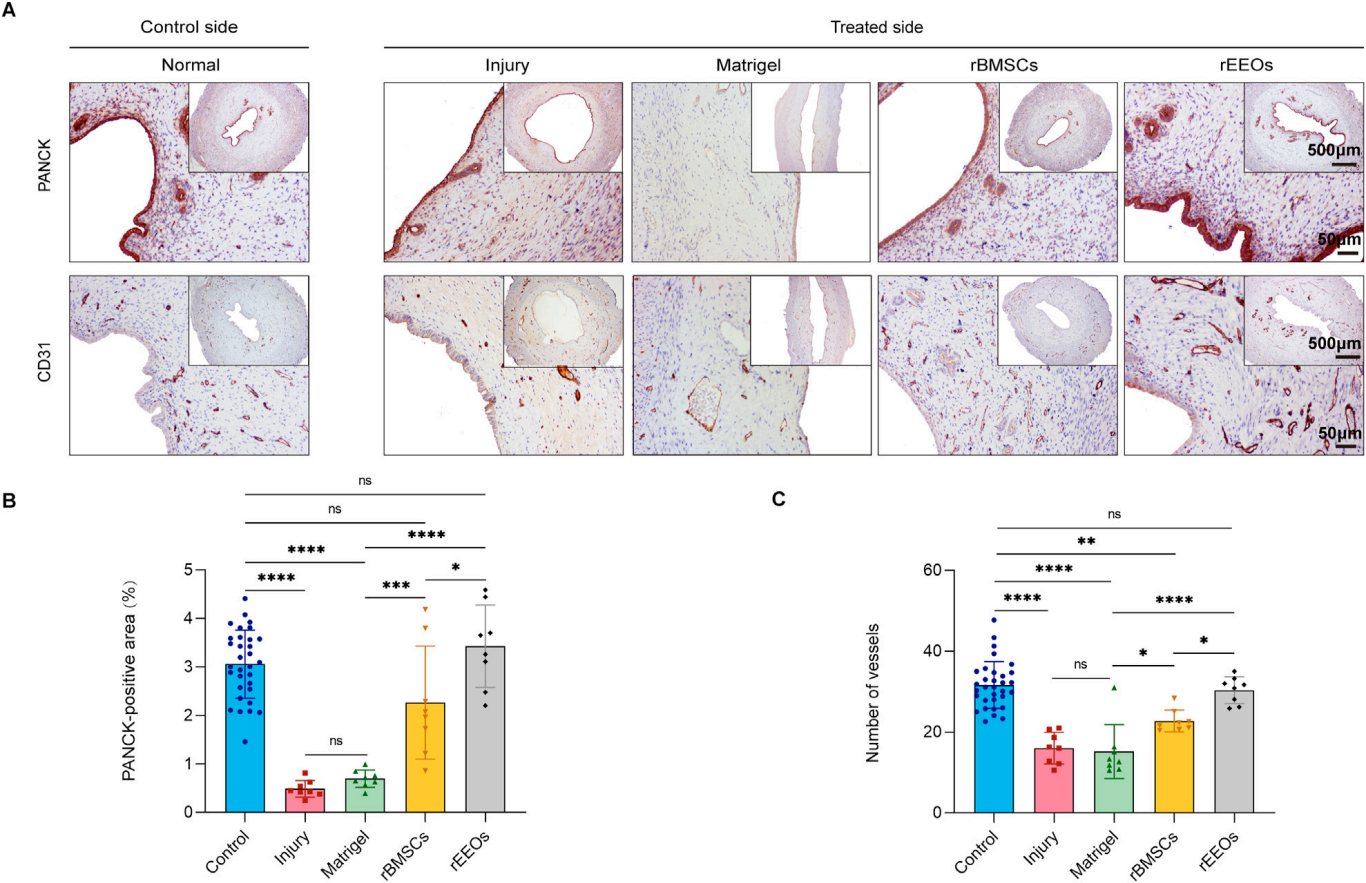
Endometrial epithelial recovery and vascular regeneration after 28 days of various treatments. **(A)** IHC staining, including PANCK and CD31, in consecutive sections of the uterus after 28 days of various treatments. Insets display overview images with lower magnification. **(B–C)** Statistical analysis of PANCK-positive areas, and the count of neovascularisations (CD31) through normalized changes after 28 days of different treatments. Control group (n = 32), Injury group (n = 8), Matrigel group (n = 8), rBMSCs group (n = 8) and rEEOs group (n = 8). The data are presented as the mean ± SD and analyzed using one-way ANOVA with Bonferroni correction.

Epithelial regeneration and sub-endometrial blood flow are essential for restoring fertility (Evans et al., 2016; Kim et al., 2010). Epithelial integrity was assessed using PANCK staining to assess the effect of rEEOs on the epithelial restoration of the injured endometrium in various treatment groups. PANCK expression in the epithelium showed progressive improvement in the rEEOs group (Early, 2.8% ± 0.7%; Mid, 3.6% ± 1.1%; Late, 3.4% ± 0.8%) and rBMSCs group (Late, 1.4% ± 0.6%; Mid, 2.1% ± 0.7%; Late, 2.3% ± 1.2%) compared to the Injury group (Early, 0.5% ± 0.1%; Mid, 0.6% ± 0.3%; Late, 0.5% ± 0.2%) and Matrigel group (Early, 0.5% ± 0.4%; Mid, 0.8% ± 0.4%; Late, 0.7% ± 0.2%) (Figures 3A, B; Supplementary Figure S4D, E; Supplementary Figure S5D, E). The restoration of epithelial integrity was significantly improved in the rEEOs group compared to the rBMSCs group at days 28 post-transplantation (Figures 3A, B). We then examined number of vessels using IHC staining for CD31. Compared with the Injury group (Early, 10.3 ± 3.7; Mid, 12.3 ± 2.7; Late, 16.0 ± 3.9) and the Matrigel group (Early, 14.3 ± 4.5; Mid, 14.4 ± 1.9; Late, 15.2 ± 6.7), vascular density in the rEEOs group (Early, 26.2 ± 5.5; Mid, 28.8 ± 12.8; Late, 30.4 ± 3.3) and rBMSCs group (Early, 22.4 ± 6.3; Mid, 20.2 ± 3.5; Late, 22.8 ± 2.7) was markedly increased on days 7, 14, and 28 (Figures 3A, C; Supplementary Figure S4D, F; Supplementary Figure S5D, F). Notably, vascular regeneration was significantly better in the rEEOs group than in the rBMSCs group on day 28 (Figures 3A, C). These findings suggest that rEEO implantation significantly contributed to reconstructing the injured endometrium.

### rEEO transplantation restores fertility in rats with endometrial injury

**FIGURE 4:**
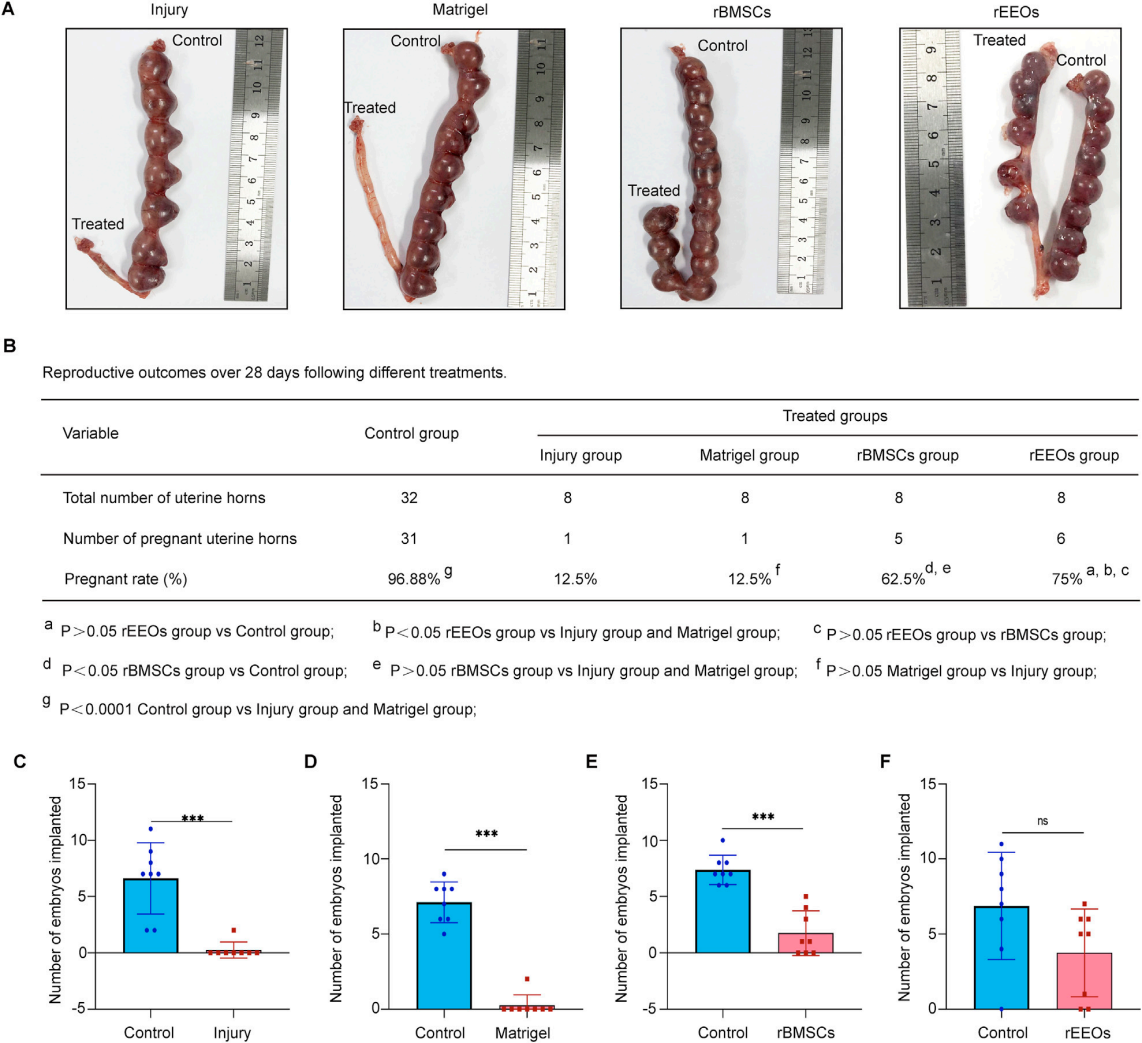
Pregnancy outcome among groups with different treatments. **(A)** Gross images displaying pregnant uteri in various treatment groups after endometrium reconstruction. **(B)** Pregnancy rates in each group. The pregnancy rate was calculated by the number of pregnant uterine horns divided by the total number of uterine horns in each group. The data are compared using the chi-square and Fisher’s precision probability tests. **(C–F)** Number of embryos implanted in each group. Each group comprised eight rats. The data are compared using the Mann–Whitney *U* test.

Fertility within the same rat was examined to evaluate the effect of rEEO transplantation on fertility recovery in rats with endometrial injury. After endometrial injury, the right uterine horn of each rat was treated while the contralateral left uterus served as a control. Gross images of the uterus were captured on day 18 of gestation (Figure 4A). A pronounced decline in the pregnancy rate was observed in the injury (*p* < 0.0001) and Matrigel groups (*p* < 0.0001) compared to the control group (Figure 4B). In contrast, uterine horns treated with rEEOs (*p* < 0.05, compared to the Injury and Matrigel groups; *p* > 0.05, compared to the control group) exhibited higher pregnancy rates (Figure 4B). Moreover, the number of embryos implanted in the injured and non-injured uterine horns of the same rat exhibited significant differences (*p* < 0.001) (Figure 4C). rEEO transplantation demonstrated a significant effect on fertility restoration (*p* > 0.05), while Matrigel (*p* < 0.001) and rBMSC (*p* < 0.001) transplants did not show a similar effect (Figures 4D–F). These findings suggest that rEEO transplantation significantly enhanced fertility restoration in rats with endometrial injury, surpassing the effects of rBMSCs transplantation.

### Differential gene expression profiles between rEEOs and rBMSCs

**FIGURE 5:**
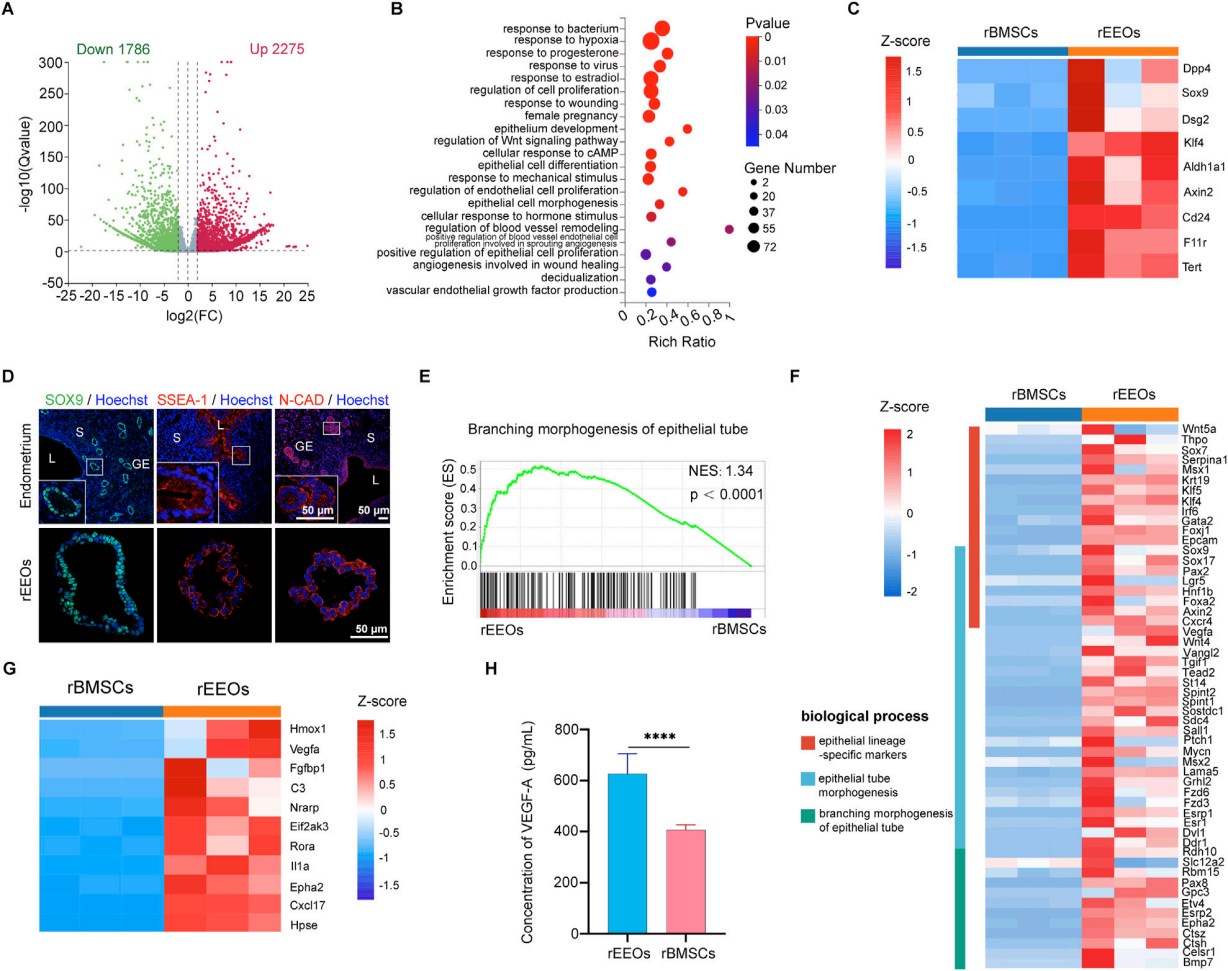
RNA-seq-based transcriptome profiles of rEEOs and rBMSCs. **(A)** Volcano plot highlighting differentially expressed genes in rEEOs and rBMSCs identified using RNA-seq. Genes depicted in green and red dots exhibit significant differential expression between rEEOs and rBMSCs (*q* < 0.05). Genes not significantly differentially expressed are shown in grey. **(B)** Enriched GO biological processes among the upregulated genes in rEEOs compared to rBMSCs (*p* < 0.05). The color key on the right denotes the significance level, with the most enriched GO categories highlighted in red. Circle size corresponds to the frequency of the GO term. **(C)** Heatmap illustrating the differential expression of genes linked with endometrial epithelial stem or progenitor cells between rEEOs and rBMSCs. TPM values of each gene across various samples were normalized to Z-scores. **(D)** Representative images of IF staining for SOX9, SSEA-1, and N-CAD in the endometrium and rEEOs. Insets highlight higher magnification images, with magnified regions marked by white squares. **(E)** GSEA plot demonstrating the enrichment of genes associated with epithelial tube morphogenesis (n = 175) in rEEOs compared to rBMSCs. NES represents the normalized enrichment score. **(F)** Heatmap displaying the genes differentially regulated in physiological functions between rEEOs and rBMSCs. The TPM values of each gene across various samples were normalized to Z-scores. **(G)** Heatmap illustrating the differential expression of genes linked with angiogenesis between rEEOs and rBMSCs. TPM values of each gene from different samples were normalized to Z-scores. **(H)** Analysis of VEGF-A levels in the rEEO and rBMSC supernatant using ELISA (n = 3). The data are presented as the mean ± SD. They were compared using the independent-samples *t*-test.

A comparative gene expression analysis using bulk RNA-seq was conducted to explore the molecular mechanisms underlying the restoration of damaged endometrium using rEEOs and distinguish the differences compared to rBMSCs. Overall, 4061 genes were differentially expressed (|Log_2_FC| > 2, *q* < 0.05) with 1786 and 2,275 genes downregulated and upregulated, respectively, in rEEOs compared to rBMSCs (Figure 5A; Supplementary Table S2). The GO analysis revealed that upregulated genes in rEEOs were associated with hormone responsiveness, epithelial development, angiogenesis, and wound healing (Figure 5B; Supplementary Table S3). rEEOs exhibited significantly higher expression levels of genes linked to endometrial epithelial stem/progenitor cells, including *Axin2*, *Sox9*, *Aldh1a1,* and *Tert,* compared to rBMSCs (Figure 5C; Supplementary Table S2). Similarly, IF staining further illustrated broadly expression of endometrial epithelial stem/progenitor markers, including SOX9, SSEA-1, and N-CAD, within endometrium and rEEOs (Figure 5D). GSEA also indicated the enrichment of genes involved in epithelial tube morphogenesis in rEEOs (Figure 5E; Supplementary Table S4). rEEOs exhibited a significant increase in the expression of epithelial lineage-specific markers, including *Krt19*, *Foxj1*, *Epcam*, *Sox17*, *Lgr5* and *Foxa2,* compared to rBMSCs (Figure 5F). Given the crucial role of angiogenesis in endometrial repair and regeneration, RNA-seq analysis revealed that several genes associated with angiogenesis are expressed in rEEOs, including *Hmox1*, *Vegfa*, *Fgfbp1* and *Cxcl17*. These genes can contribute to the enhancement of endometrial vasculature regeneration to a certain extent (Figure 5G; Supplementary Table S2). Furthermore, ELISA analysis demonstrated increased VEGF-A secretion by rEEOs, a potent vascular regeneration inducer (Figure 5H). The disparities observed in the gene expression profiles indicate the presence of numerous epithelial tube morphogenesis-related genes in rEEOs. Moreover, genes associated with endometrial epithelial stem/progenitor cells and the facilitation of vascular regeneration are also expressed in rEEOs. These characteristics may potentially underpin their robust endometrial regenerative potential.

## Integration and functional contribution of rEEOs to injured endometrium

**FIGURE 6:**
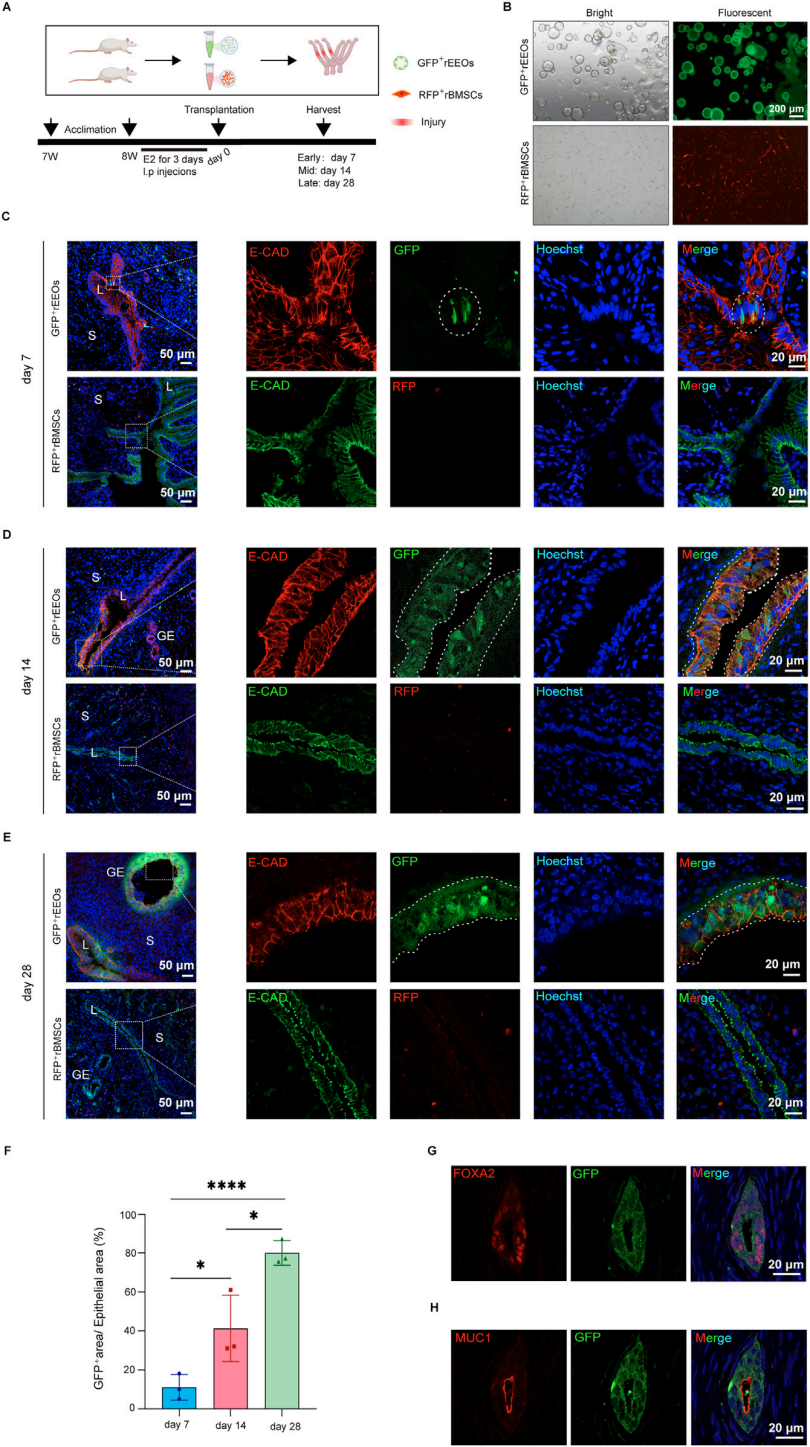
rEEO integration into the injured endometrium after transplantation. **(A)** Depiction of the experimental protocol used to track the fate of rEEOs *in vivo.* **(B)** Visual representation of GFP-labeled rEEOs and RFP-labeled rBMSCs. **(C–E)** Depictions of the recipient uterus on days 7 **(C)**, 14 **(D)**, and 28 **(E)** post-transplantation. Higher magnification views of the areas in the dotted squares are presented on the right. GFP-labeled cells integrated into the receptor endometrial are marked with white dotted lines. **(F)** The proportion of GFP-positive area to the epithelial area (labeled with E-cad) after 7, 14, and 28 days of rEEOs transplantation (100× field of view) (n = 3). The data are presented as the mean ± SD and analyzed using one-way ANOVA with Bonferroni correction. **(G–H)** Illustration of IF staining for GFP, FOXA2, and MUC1 in the recipient uterus. L, lumen; S, stroma; GE, glandular epithelium.

The fate of the transplanted rEEOs was tracked to explore the mechanism of endometrial regeneration using rEEOs. HE staining of the uterus on the initial postoperative day revealed a considerable absence of the endometrial lumen in the epithelium, along with some damaged glands (Supplementary Figure S6A). GFP-labeled rEEOs and RFP-labeled rBMSCs were transplanted into the uteri of rats with endometrial injury to observe whether these cells could integrate into the recipient endometrial tissue (Figures 6A, B). Fluorescence imaging confirmed the successful transplantation of both cell types into the uterine cavity (Supplementary Figure S6B). Moreover, IF staining for the epithelial marker E-CAD confirmed the presence of cells derived from GFP-labeled rEEOs in the luminal epithelium of the endometrium 7 days post-transplantation, despite the low number of GFP^+^ cells (Figure 6C). Additionally, we observed the integration of these cells in multiple locations (Supplementary Figure S6C). On day 14 post-transplantation, patches of GFP-labeled cells representing clones originating from the transplanted rEEOs were observed (Figure 6D). On day 28 post-transplantation, GFP-labeled glands were present beneath the luminal epithelium, and multiple GFP-labeled crypts were observed (Figure 6E; Supplementary Figure S6D, E). Statistical analysis of GFP^+^ cells at different time points after rEEOs transplantation revealed a significant increase in the proportion of GFP^+^ cells with prolonged transplantation time (Figure 6F). This suggested that the cells derived from rEEOs possess a certain degree of self-renewal ability. Nevertheless, we observed only a few infiltration of RFP^+^ rBMSCs at the mesenchymal sites of the endometrium transplanted with RFP^+^ rBMSCs. However, we did not detect any red fluorescent signals at the epithelial sites of the endometrium (Figures 6C–E). These findings further highlighted the distinctive epithelial integration potential of rEEOs.

FOXA2 expression, a recognized marker for mature glands (Kelleher et al., 2017; Kelleher et al., 2019; Spencer et al., 2019), was examined to evaluate the functionality of rEEOs-derived glands *in vivo.* IF analysis confirmed FOXA2 expression in glands derived from GFP-labeled rEEOs (Figure 6G). Moreover, these rEEO-derived glands expressed the secretory epithelial marker MUC1 (Figure 6H). These results indicate that transplanted rEEOs have the ability to integrate into the epithelium and subsequently undergo differentiation into endometrial glandular tissues within the recipient rats. Furthermore, these glands express characteristic markers of mature glands and have endometrial secretory characteristics.

## Discussion

The adult human endometrium comprises two distinct anatomical regions: the upper functionalis and the deeper basalis. The functional layer is shed, whereas the permanent basal layer regenerates a new functional layer according to fluctuating estrogen and progesterone levels (Evans et al., 2016). Severe trauma can hinder the regenerative capacity of the endometrium, consequently impairing embryo implantation (Yu et al., 2008). However, clinical treatments for endometrial injury remain limited (Lin et al., 2013). Recently, 3D-cultured organoids have been used as promising regenerative tools (Lancaster and Knoblich, 2014; Kim et al., 2023). For example, colon organoids transplanted into damaged colons re-established epithelial structure and integrity, serving as a potential treatment for gastrointestinal diseases (Yui et al., 2012; Fordham et al., 2013; Sugimoto et al., 2018; Jee et al., 2021). Furthermore, cortical organoids have been successfully integrated into damaged brain cavities for cortical repair, demonstrating potential in treating neuronal conditions (Revah et al., 2022; Wilson et al., 2022; Jgamadze et al., 2023; Zheng et al., 2023). Organoids have also been used to treat bile duct reparation (Sampaziotis et al., 2021). Similarly, the present study highlights that EEO allografts effectively repair damaged endometrium and reinstate fertility by integrating into the injured endometrium of recipient rats. This research offers compelling evidence supporting the use of EEO transplantation to promote endometrial regeneration within reproductive medicine.

Although EEOs have certain effects on endometrial repair, the precise molecular mechanisms driving these effects remain unclear (Jiang et al., 2021; Zhang et al., 2022; Xu et al., 2023). Research examining the mechanisms behind organoid efficacy in tissue repair has primarily concentrated on the pluripotent traits of stem and progenitor cells within these organoids (Lancaster and Knoblich, 2014; Boretto et al., 2017; Turco et al., 2017). Jee et al. highlighted the critical role of Lgr5^+^ progenitor cells within colon organoids and the therapeutic benefits (Jee et al., 2021). Furthermore, lung organoid cells maintain their progenitor cell function post-transplantation (Louie et al., 2022). The epithelial stem or progenitor cell pool, primarily within glands and luminal crypts, can differentiate into glandular cells and facilitate endometrial repair after injury (Jin, 2019; Seishima et al., 2019; Syed et al., 2020). Data from single-cell RNA-seq conducted by two research groups and the bulk RNA-seq data of this study have identified potential progenitor cell types in the EEOs (Fitzgerald et al., 2019; Garcia-Alonso et al., 2021). Similarly, Jin et al. recently revealed a bipotent uterine epithelial stem cell population capable of initiating epithelial regeneration using a CK19-driven Cre reporter mouse model (Jin, 2019). This population generates a complete endometrial epithelial lineage, encompassing surface luminal epithelia and glands embedded in the stroma (Jin, 2019). *In vivo* genetic lineage tracing studies revealed that removing long-lived bipotent Axin2^+^ epithelial progenitor cells results in impaired endometrial regeneration (Syed et al., 2020). In the present study, endometrial injury was induced using EDTA intrauterine instillation and repeated scratching, mimicking a model where the endometrial glands are not eliminated. Despite endometrial regeneration partly relying on residual glands post-injury, distinct GFP^+^ cells were observed in the epithelial area of the recipient endometrium after GFP-labeled rEEO transplantation. Moreover, these GFP^+^ cells generated larger clones within the epithelial crypts and eventually developed into functional glands. These findings offer convincing evidence that rEEO-derived cells integrating into the injured endometrial tissue of the recipient may represent bipotent uterine epithelial stem cells capable of producing endometrial luminal epithelium and glands (Jin, 2019). However, further characterization of this cell population within EEOs and identifying dependable markers are essential. Analysis of bulk RNA-seq data on EEOs and BMSCs revealed that EEOs express more stem cell markers, including *Axin2*, *Tert*, *Aldh1a1*, et. And research has shown that Axin2^+^ cells play a critical role in the development of endometrium (Syed et al., 2020). Additionally, upregulated genes in EEOs are involved in the regulation of the Wnt signaling pathway. Studies have shown that the Wnt signaling pathway plays a crucial role in the self-renewal process of EEOs (Boretto et al., 2017), and regulate the development and regeneration of the endometrial epithelium (Seishima et al., 2019). This suggests that the Wnt signaling pathway may be a potential molecular mechanism driving the fusion and differentiation of EEOs into receptive endometrial tissue. Furthermore, endometrial repair entails various intricate processes, and while EEO integration and differentiation are essential, they likely only partially constitute the complex mechanisms involved.

Stem cell-based therapies, particularly using BMSCs, can rejuvenate endometrial tissue. Paracrine signaling represents the primary mechanism driving the effectiveness of mesenchymal stem cells (MSCs) (Syed et al., 2020; Azizi et al., 2018; Cousins et al., 2021a; Song et al., 2021). The study examined the secretion levels of VEGF-A in EEOs and BMSCs. VEGF-A plays a critical role in angiogenesis through the VEGF-KDR/Flk-1 signaling pathway (Kliche and Waltenberger, 2001). It promotes endothelial cell proliferation and migration and regulates vascular permeability (Kliche and Waltenberger, 2001). The results showed that EEOs secrete more VEGF-A than BMSCs, suggesting that EEOs may also promote endometrial repair and regeneration through some paracrine mechanisms. However, extensive literature suggests that BMSCs promote vascular regeneration and anti-fibrosis through paracrine secretion (Azizi et al., 2018; Cousins et al., 2021a; Song et al., 2021). Whether these mechanisms also exist in EEOs needs further exploration.

The results also revealed a discrepancy in integrating rBMSC- and rEEO-derived labeled cells after transplantation into the endometrial epithelium of the recipient. While some RFP-labeled rBMSCs infiltrated the endometrium, GFP-labeled cells from rEEOs exhibited efficient integration into the recipient endometrial epithelium. This finding aligns with that of previous studies indicating that MSCs can infiltrate the interstitium (Cervelló et al., 2015; Liu et al., 2018) and cannot directly differentiate into endometrial cells to facilitate regeneration and renewal (Syed et al., 2020; Ong et al., 2018). Transplanting endometrial epithelial progenitor cells exhibits greater therapeutic promise than endometrial MSCs for regenerating injured endometrial tissue (Darzi et al., 2016; He et al., 2022). However, prolonged *in vitro* expansion of two-dimensional cultured endometrial epithelial progenitor cells faces challenges due to a substantial increase in apoptosis (He et al., 2022). Our research and other studies have demonstrated that rEEOs reliably contain many epithelial progenitor cells, surmounting the challenges faced by two-dimensional cultured epithelial cells that lack stability for prolonged *in vitro* expansion (Lancaster and Knoblich, 2014; Turco et al., 2017; Fitzgerald et al., 2019). Consequently, micro 3D-cultured rEEOs exhibit enhanced regenerative capabilities for damaged endometria compared to conventional two-dimensional cultured stem cells.

Modern medicine extensively explores the potential for replacing impaired or dysfunctional tissues through homologous transplantation. However, identifying the source of the transplanted tissue remains challenging (Kim et al., 2023). Various organoids derived from human body parts, such as the gut, brain, and retina, have been effectively used to repair damaged organs (Yui et al., 2012; Fordham et al., 2013; Sugimoto et al., 2018; Jee et al., 2021; Revah et al., 2022; Wilson et al., 2022; Jgamadze et al., 2023; Zheng et al., 2023; McLelland et al., 2018). Therefore, a promising approach involves producing genetically identical EEOs using stem cells obtained from the endometrium of the patient. These engineered organoids can be transplanted into the uterine cavity to rejuvenate the impaired endometrium. This strategy circumvents potential immune rejection associated with alternative cell sources, presenting significant potential for organ regeneration. However, it is worth noting that the use of Matrigel as a biological product presents certain limitations in terms of its promotion and application (Kleinman and Martin, 2005). Therefore, exploring alternative culture matrices and biological scaffolds is crucial for further optimization. Furthermore, humans are among the very few species that menstruate (Evans et al., 2016), whether the role of organoid therapy for endometrial damage is affected by menstruation must be further explored. Despite the need for ongoing refinement, our study suggests that the *in vitro* expansion and transplantation of EEOs may offer a potential therapeutic option to restore fertility.

In summary, EEOs offer a transformative approach to address the challenges of endometrial trauma. Their remarkable regenerative potential, supported by their integration into the recipient endometrium and their differentiation into functional glandular epithelial cells, holds promise for the restoration of damaged endometrium. As we venture into the future, the concept of utilizing patient-specific EEOs for transplantation emerges as a tantalizing prospect, unifying regenerative medicine and personalized therapy. Although challenges remain, The present study offers compelling evidence to support the clinical application of EEOs for endometrial repair, consequently contributing to advancing therapeutic options in reproductive medicine.

## Conclusion

Long-term amplified rEEOs were successfully cultivated in the present study. *In vivo* experiments indicated that rEEOs exhibited superior performance compared to rBMSCs in repairing damaged endometrial tissue and restoring fertility in rats with injured endometrium. The remarkable regenerative capability of rEEOs is evidenced by their integration into the recipient endometrium and transformation into functional glandular epithelial cells. This study significantly contributes to reproductive medicine by introducing EEOs as a potential breakthrough in restoring fertility by repairing impaired endometrial tissue. These findings pave the way for further research and development of EEO-based therapeutic interventions, offering hope for individuals struggling with endometrial trauma-related infertility.

## Data Availability

The sequencing data generated in this study have been deposited in the Gene Expression Omnibus database under accession code GSE269747.

## Abbreviations

BMSCs, bone marrow mesenchymal stem cell; cAMP, cyclic adenosine monophosphate; CO_2_, carbon dioxide; 3D, three-dimensional; EEOs, endometrial epithelial organoids; ELISA, Enzyme-linked immunosorbent assay; E2, oestrogen; FBS, Foetal bovine serum; GFP, green fluorescent protein; GSEA, Gene Set Enrichment Analysis; HE, Haematoxylin-Eosin; IF, Immunofluorescence; IHC, Immunohistochemistry; IUA, Intrauterine adhesions; IVIS, *in vivo* imaging system; Masson, Masson’s trichrome; OCT, optimal cutting temperature compound; PAS, Periodic Acid-Schiff; PBS, phosphate buffered saline; P4, progesterone; rBMSCs, rat bone marrow mesenchymal stem cell; rEEOs, rat endometrial epithelial organoids; RFP, red fluorescent protein; SD rat, Sprague-Dawley rat; SD, standard deviation; RNA-seq, RNA-sequencing; TPM, transcripts per kilobase of exon model per million mapped reads; VEGF, vascular endothelial growth factor.
