# Supplementary material for: Engraftment of self-renewing endometrial epithelial organoids promotes endometrial regeneration by differentiating into functional glands in rats
Source: Front Bioeng Biotechnol. 2024 Dec 11;12:1449955. doi: 10.3389/fbioe.2024.1449955 (PMC11668608; doi:10.3389/fbioe.2024.1449955)
Supplement: Supplementary file 5 [file DataSheet1.docx]

Supplementary Material

**Engraftment of self-renewing endometrial epithelial organoids promotes endometrial** **regeneration by differentiating into** **functional glands in rats**

Xiaona Lin*, Mengying Liu*，Yana Ma, Jingjing Qian

*** Correspondence:** Xiaona Lin*: linna73@zju.edu.cn or Mengying Liu *: [3322074@zju.edu.cn](mailto:3322074@zju.edu.cn)

# Supplementary Figures


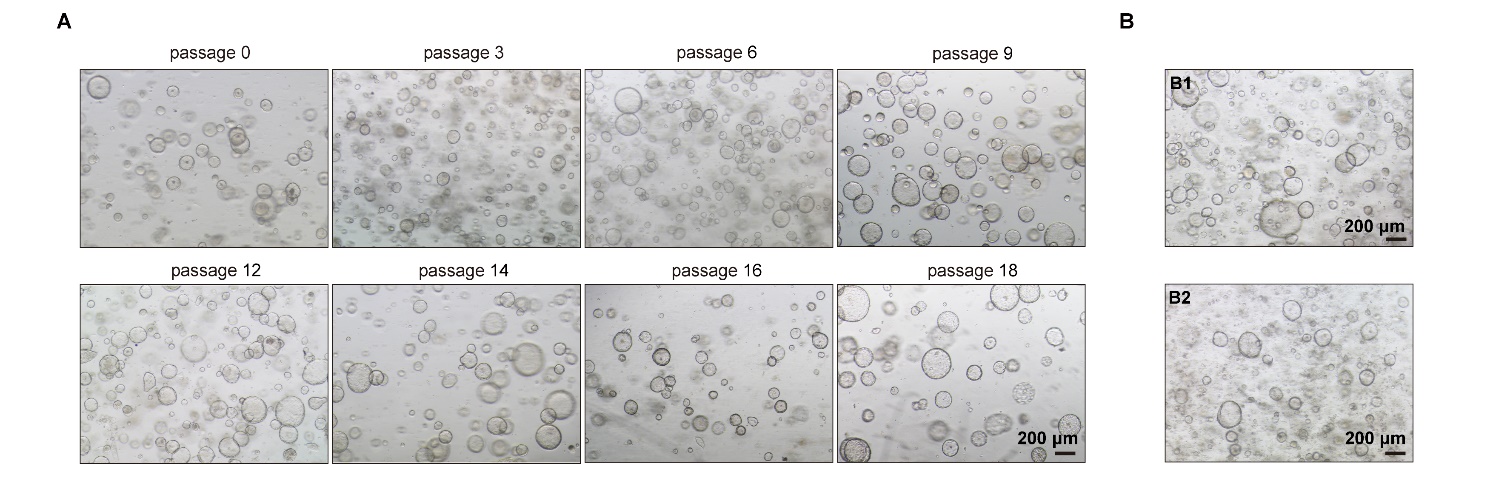


**Supplementary Figure S1. Long-term expansion and passaging capability of rEEOs.** (A) Depiction of representative images of rEEOs at the indicated passage numbers. (B) Depiction of representative images of rEEOs at passage 12 before being frozen (B1) and after being thawed (B2).


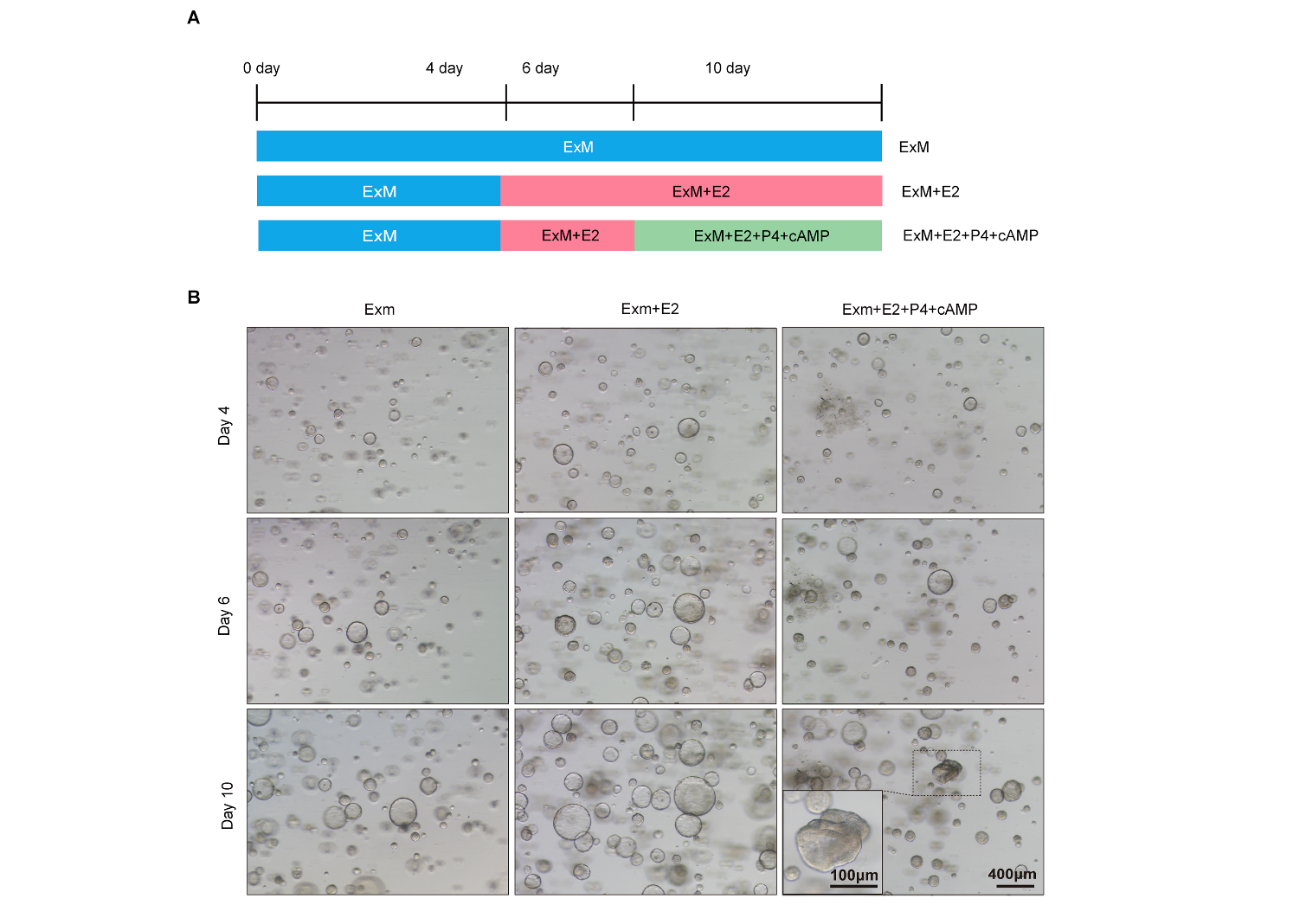


**Supplementary Figure 2. Alteration in rEEO morphology induced in response to hormones.** (A) Description of the protocol outlining the hormone stimulation of rEEOs. (B) Depiction of morphological alterations of rEEOs in response to various hormone treatments. The inset shows folding and wrinkling rEEOs at higher magnification.


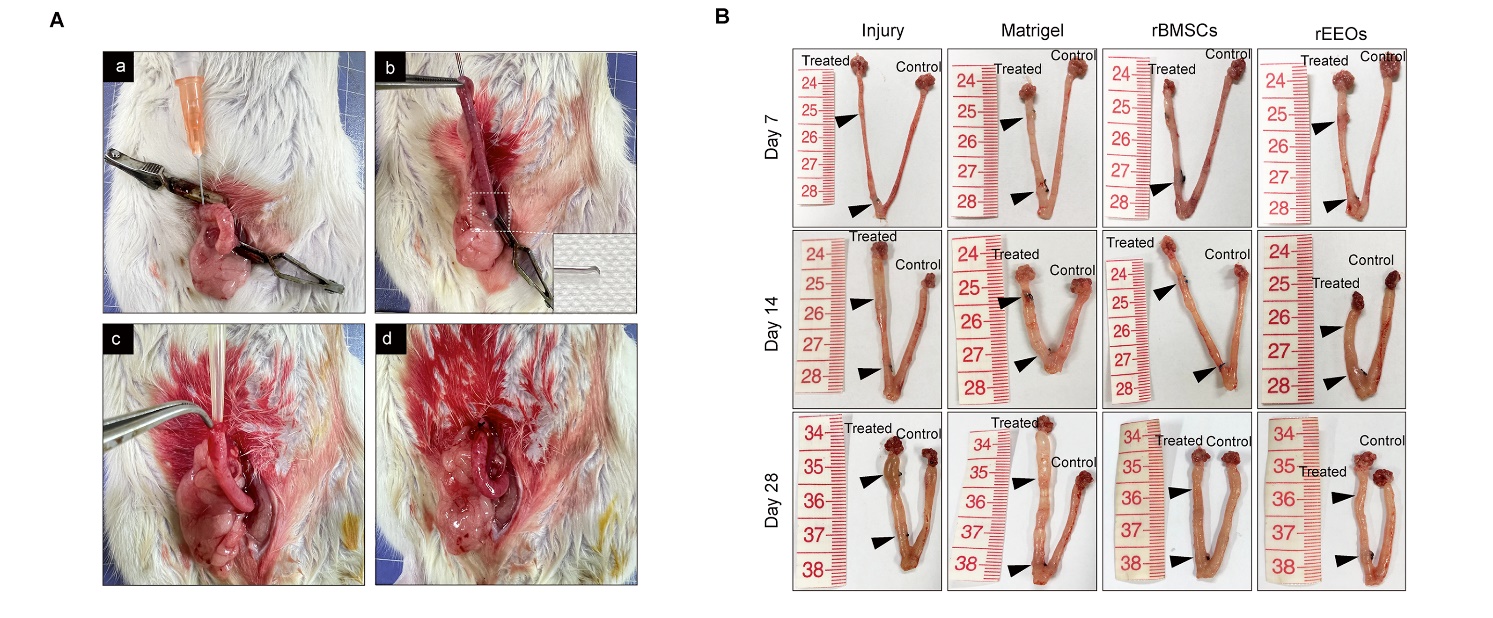


**Supplementary Figure 3. Establishment of endometrial injury model and the uterine horns receiving different treatments.** (A) Description of the steps involved in inducing endometrial injury: (a) 250 mM EDTA perfusion of the uterine horn. (b) Repeated scratching of the uterine cavity with a spatula. The inset shows the spatula at higher magnification. (c) Transplantation of cells into the uterus. (d) Post transplantation appearance of uterine horns. (B) Depiction of gross images of the uterine horns at 7, 14, and 28 days after different treatments. The black arrows indicate the extent of the injured area.


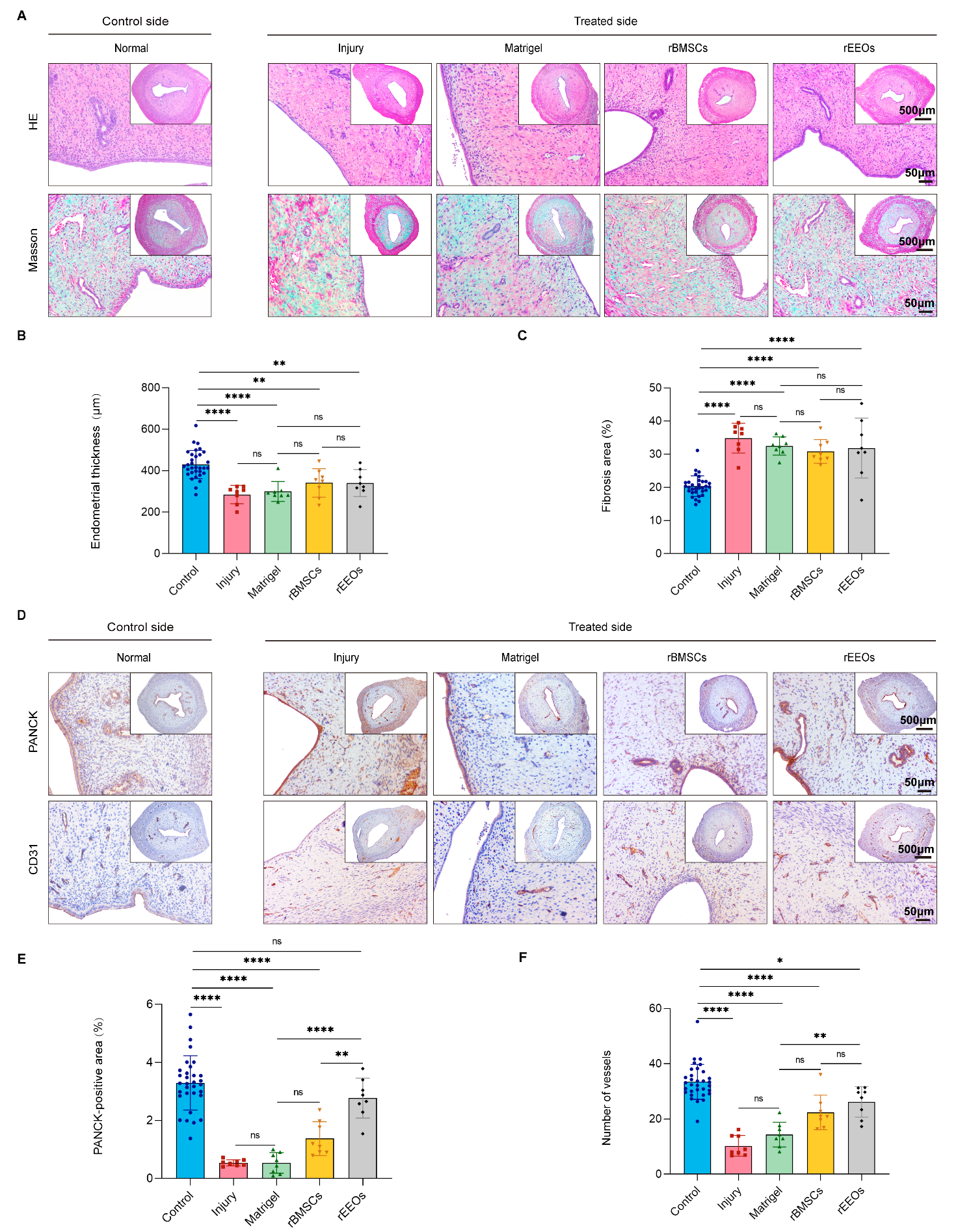


**Supplementary Figure 4. Recovery of injured endometrium after 7 days of different treatments.** (A) HE and Masson staining of consecutive sections of the uterus after 7 days of distinct treatments. Insets display overview images at lower magnification. (B–C) Statistical analysis of the normalized changes in endometrial thickness and fibrosis area after 7 days of diverse treatments. (D) IHC staining, including PANCK and CD31, in consecutive sections of the uterus following 7 days of various treatments. Insets display overview images with lower magnification. (E–F) Statistical analysis of PANCK positive areas, and the number of neovascularisations (CD31) through normalized changes after 7 days of different treatments. Control group (n=32), Injury group (n=8), Matrigel group (n=8), rBMSCs group (n=8) and rEEOs group (n=8). The data are presented as the mean ± SD and analyzed using one-way ANOVA with Bonferroni correction. **p* < 0.05; ***p* < 0.005; ****p* < 0.001; *****p* < 0.0001; ns, no significance.


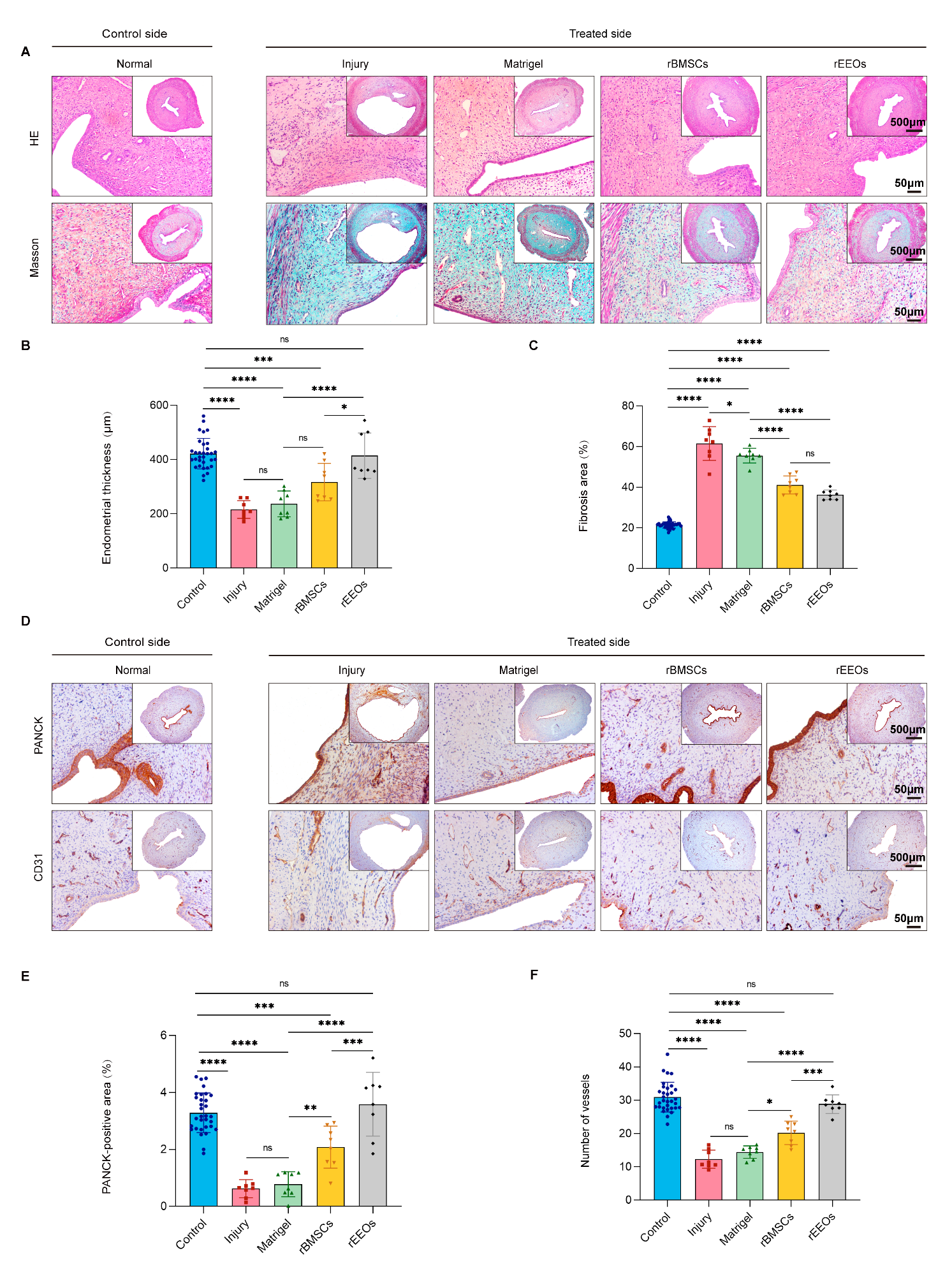


**Supplementary Figure 5. Recovery of the injured endometrium after 14 days of various treatments.**  (A) HE and Masson staining of consecutive sections of the uterus after 14 days of distinct treatments. Insets display overview images at lower magnification. (B–C) Statistical analysis of the normalized changes in endometrial thickness and fibrosis area following 14 days of diverse treatments. (D) IHC staining, including PANCK and CD31, in consecutive sections of the uterus following 14 days of various treatments. Insets display overview images with lower magnification. (E–F) Statistical analysis of PANCK positive areas, and the number of neovascularisations (CD31) through normalized changes after 14 days of different treatments. Control group (n=32), Injury group (n=8), Matrigel group (n=8), rBMSCs group (n=8) and rEEOs group (n=8). The data are presented as the mean ± SD and analyzed using one-way ANOVA with Bonferroni correction. **p* < 0.05; ***p* < 0.005; ****p* < 0.001; *****p* < 0.0001; ns, no significance.

**
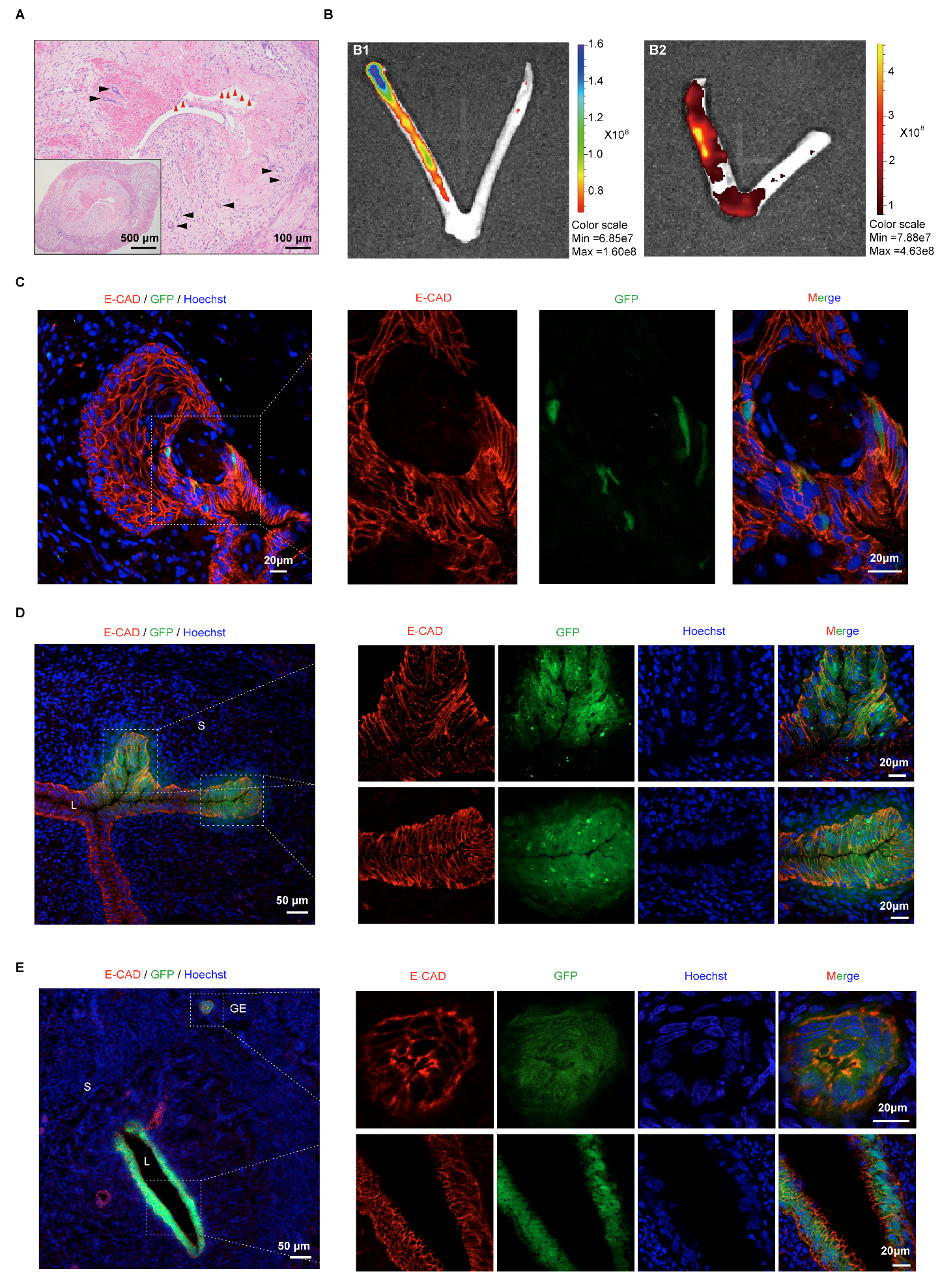
**

**Supplementary Figure 6. Tracing GFP-labeled rEEOs *in vivo*.** (A) Representative images showing HE staining of the uterus 1 day after injury surgery. Red arrows indicate defective luminal epithelium, whereas black arrows indicate residual glandular structures. (B) Fluoroscopic image of the recipient uterus at 1-day post-transplantation of GFP-labeled rEEOs (B1) and RFP-labeled rBMSCs (B2). (C) Representative images of the uterus of the rats at 7 days post-transplantation. (D–E) Representative images of the uterus of the rats at 28 days post-transplantation. Higher magnification views of the areas in the dotted squares are shown on the right. L, lumen; S, stroma; GE, glandular epithelium.
